# Changes in hemispheric dominance following targeted muscle reinnervation: a case study

**DOI:** 10.3389/fnhum.2025.1665931

**Published:** 2025-11-07

**Authors:** Toka Mootaz AboElnour, Kaitlin Fraser Wilsey, Kai Yang, Jordan Alexander Borrell, Jorge Zuniga

**Affiliations:** 1 Additive Manufacturing Laboratory, Department of Biomechanics, University of Nebraska at Omaha, Omaha, NE, United States; 2 Division of Plastic and Reconstructive Surgery, Department of Surgery, University of Nebraska Medical Center, Omaha, NE, United States; 3 Department of Occupational Therapy Education, University of Kansas Medical Center, Kansas City, KS, United States

**Keywords:** functional near infrared spectroscopy, targeted muscle reinnervation (TMR), phantom limb pain (PLP), amputation, hemodynamic response, neural plasticity, functional connectivity, hemispheric dominance

## Abstract

Phantom limb pain (PLP) after amputation is a multifaceted condition. Targeted muscle reinnervation (TMR) surgery coapts amputated nerves to motor nerves of regional muscles, closing the neuromuscular loop, enabling improved myoelectric prosthesis control and reducing PLP. Long-term effects of TMR and residual limb use have been observed; however, the short-term neural changes and their timeline are not understood. The purpose of this study was to examine the cortical changes shortly after TMR without a prosthesis, specifically the functional connectivity and hemispheric dominance during a motor task involving the affected limb. The case participant is a male 52 years old, with a left traumatic transradial amputation sustained 4 years earlier, scheduled for TMR surgery. Data was collected before and 2 months after TMR. Brain activity was recorded using functional near-infrared spectroscopy (fNIRS) while the participant performed a gross manual dexterity task (box and block test) using their phantom hand. Pain levels were assessed using a 10-point visual analog scale (VAS). Following TMR, the participant reported a VAS score of 0 and increased use of the amputated limb in daily activities. fNIRS analysis during the affected limb task showed a reduction in interhemispheric functional connectivity, prominently in the primary sensory cortex, where the average z-value decreased from 0.29 to 0.12 after TMR. In contrast, connectivity between the premotor and supplementary motor areas increased slightly, from 0.08 to 0.12. Overall, intrahemispheric correlations decreased, with opposite patterns observed across hemispheres. The largest changes occurred ipsilaterally: connectivity between the primary motor and sensory areas increased from 0.23 to 0.27, while contralaterally it decreased from 0.22 to 0.16. Conversely, connectivity between the primary motor and premotor areas increased contralaterally but decreased ipsilaterally. Hemispheric dominance calculated through the Laterality index (LI) shifted from bilateral (LI = 0.079) to ipsilateral (LI = 0.59), primarily driven by reduced activation in the contralateral primary motor cortex. These findings suggest that TMR alone can elicit measurable cortical changes in the early post-surgical period, alongside improvements in pain and functional limb use. They also support fNIRS as a non-invasive method for monitoring neural adaptation after TMR and enhance understanding of PLP mechanisms and recovery timelines.

## 1 Introduction

In the United States, 31,450 upper limb amputations occur annually, resulting in permanent functional limitation (Dillingham et al., 2002, 1998; Owings and Kozak, 1998). People with amputation commonly (87%) experience phantom limb pain (PLP) (Stankevicius et al., 2021). PLP is a localized pain with enigmatic origins and variable manifestations, tending to be more intense and constant in upper limb amputation, affecting all aspects of life, including mental health, life quality, and employment (Cole et al., 2009; Hill, 1999; Jang et al., 2011; Nikolajsen and Jensen, 2001; Padovani et al., 2015).

Targeted muscle reinnervation (TMR), a promising surgical intervention for PLP, was originally developed to improve myoelectric prosthesis control. TMR reroutes residual limb nerves to alternative muscles, preventing painful neuroma formation and closing the sensorimotor feedback loop. Surface electromyography (EMG) detects neuromuscular signals for myoelectric prosthesis control (Dumanian et al., 2019; Kuiken et al., 2009). Beyond functional benefits, TMR reduces PLP (Serino et al., 2017), and has been associated with altered functional connectivity and hemispheric dominance.

Functional connectivity, defined as the covariation of separate brain regions, provides a framework to study cortical pattern changes (Friston, 1994). Interhemispheric functional connectivity supports coordination of movement and sensory integration across hemispheres and is disrupted in stroke and amputation (Takeuchi et al., 2012). After amputation, interhemispheric sensorimotor connectivity is generally reduced compared to healthy controls (Bramati et al., 2019; Hahamy et al., 2015; Makin et al., 2013a; Zhang et al., 2018). Early evidence suggests TMR further modifies these patterns: one study reported reduced interhemispheric connectivity after TMR combined with therapy (Borrell et al., 2023b), another observed increased intrahemispheric connectivity between motor and sensory cortices, resembling healthy controls (Serino et al., 2017).

Hemispheric dominance offers an additional perspective. Functional magnetic resonance imaging (fMRI) studies use the Laterality Index (LI) to quantify asymmetry in cortical activation, a descriptive measure assessing overall hemispheric dominance (Galaburda et al., 1990; Güntürkün et al., 2020; Hutsler and Galuske, 2003; Nirkko et al., 2001; Seghier, 2008). In unimanual tasks, the sensorimotor cortex predominantly controls the contralateral body side, but amputation disrupts this balance. Reduced inhibition in the affected hemisphere leads to diffuse ipsilateral activation (Chen et al., 2013; Philip and Frey, 2014), a pattern observed in children with congenital limb deficiencies (Zuniga et al., 2021). Following TMR, cortical activity shifts back toward contralateral dominance. One study reported contralateral focused activation, resembling controls (Yao et al., 2015), while another combining TMR with individualized phantom limb therapy observed balanced activation changes, with increased activity in the primary contralateral motor areas (Borrell et al., 2023b). Following hand transplantation, cortical activity may shift back toward contralateral dominance in higher motor planning areas (Piza-Katzer et al., 2007).

Despite these advances, short-term cortical responses to TMR remain poorly defined. Early changes in hemispheric dominance and connectivity, occurring before long-term prosthesis-driven plasticity, are not well characterized (Yao et al., 2015). We address that gap by analyzing connectivity and dominance patterns in the early postoperative period. We hypothesize that TMR induces short-term cortical changes associated with pain reduction, enabling increased limb use before long-term prosthesis use-dependent plasticity and neuromuscular loop closure. Specifically, we hypothesize changes in interhemispheric functional connectivity after TMR, increases in intrahemispheric connectivity between the primary motor (M1) and primary sensory (S1) cortices in the contralateral hemisphere during task performance. Additionally, pre-TMR dominance will be biased away from the contralateral side, shifting post-TMR toward increased contralateral activation, with higher motor planning regions showing stronger activity first.

## 2 Materials and methods

### 2.1 Patient recruitment

The participant was referred by the performing surgeon. Inclusion criteria included the ability to follow task instructions, move the phantom limb, and complete both pre- and post-surgical data collection. Due to limited contraindications against fNIRS and the rarity of local TMR cases, there were no further contraindications beyond major upper limb motion limitation. The participant was a 52-year-old right-handed man, self-reported ambidextrous, a retired painter, with hypertension and diabetes, 6′2″ tall, and weighed 275 lbs. On July 4, 2020, he sustained a traumatic left transradial amputation, followed by recurrent neuroma formation and chronic pain requiring multiple excision surgeries. In March 2024, he underwent TMR to treat neuromas and enable myoelectric prosthesis use. The procedure involved excising median and ulnar nerve neuromas and transferring the nerves to the motor branches of multiple muscles at the elbow level. The median nerve was transferred to the motor branches of the pronator quadratus and the flexor digitorum superficialis muscles, and the ulnar nerve to the flexor carpi ulnaris muscle.

### 2.2 Experimental setup

**Figure 1:**
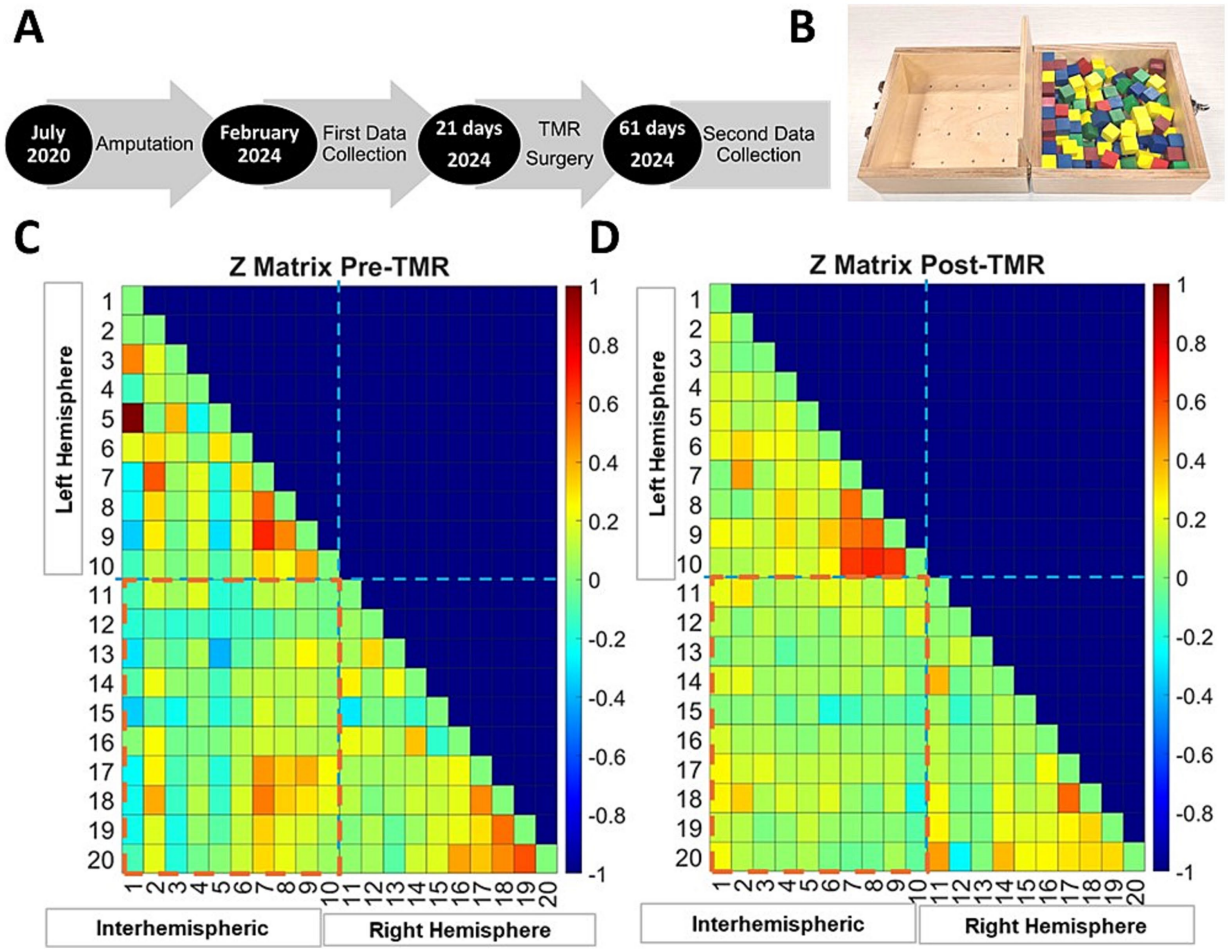
Timeline of surgeries and data collection **(A)**. Box and blocks test, the task during which the fNIRS data was collected **(B)**. Fisher-transformed correlation matrices showing functional connectivity strength in both hemispheres during the movement task. Intrahemispheric connections are displayed for the left and right hemispheres, and interhemispheric connections are indicated across the red dashed line for before TMR **(C)** and after **(D)**.

Data was collected across two visits and supplemented with the surgeon’s notes (Figure 1A). The first visit occurred in February 2024, followed by the TMR procedure 21 days later in March. The second visit was in May 2024, 61 days after TMR; no prosthesis was used during that time. Each visit lasted 2 h; cortical activity was recorded while the participant performed a motor task. The protocol began and concluded with a 3-min rest, with tasks with the non-affected and then affected limb in between. Tasks involved 30 s of rest and 1 min of activity, repeated three times; analysis hereafter will be focused on the affected limb only. Post-TMR, Oxycodone was prescribed for 3–5 days for pain. The participant provided written informed consent, and the study was approved by the University of Nebraska Institutional Review Board.

### 2.3 Experimental protocol

#### 2.3.1 Gross manual dexterity task motor imagery

The box and block test (Figure 1B), a measure of gross manual dexterity (Mathiowetz et al., 1985), was used as the motor task performed during cortical activity recording, which involves moving as many 1-inch blocks as possible from one side of the box to the other, across a divider, in 1 min. For the phantom limb despite lacking a physical hand, he attempted to perform each step to move the blocks, picking up the block, transferring it across the divider, and releasing it, without a prosthesis (Raffin et al., 2016; Scaliti et al., 2020). The unaffected limb was tested first for task familiarization.

#### 2.3.2 Pain level

Pain and medication use were documented via interviews on data collection days and medical records. Pain was self-reported on a 10-point visual analog scale (1 = no pain, 10 = very painful) (Price et al., 1983).

#### 2.3.3 Functional Near-Infrared Spectroscopy (fNIRS)

Functional Near-Infrared Spectroscopy (fNIRS) is a non-invasive method for measuring cortical activity a few centimeters below the surface (Pinti et al., 2020), through emitting near-infrared light at two wavelengths and detecting reflected signals to measure hemodynamic responses via changes in oxygenated (HbO2) and deoxygenated (HbR) hemoglobin (Scholkmann et al., 2014). In contrast to the gold standard fMRI, fNIRS is portable, movement-tolerant, and safe, making it suitable for active tasks and clinical populations contraindicated to fMRI (Scholkmann et al., 2014). Recent work demonstrated its utility for monitoring cortical activity, functional connectivity, and hemispheric activation in this group (Bai et al., 2020; Borrell et al., 2024, 2023a, 2023b; Karumattu Manattu et al., 2023; Li et al., 2023; Shen et al., 2025).

fNIRS data were collected using the NIRSport 2 system (NIRx Medical Technologies, LLC, Berlin, Germany), sampled at 8 Hz with wavelengths of 760 and 850 nm. The cap included 15 detectors, 16 sources, and 8 short-separation channels to filter superficial noise (Zhang et al., 2021). It was positioned over the sensorimotor cortex according to the international 10–20 system, covering the C3 and C4 landmarks to target upper limb motor activity (Nishiyori et al., 2016; The International Federation of Clinical Neurophysiology, n.d.).

### 2.4 Analysis

Analysis focused on the affected limb task. Trials with motion artifacts were discarded and repeated. Short separation channels were used to filter superficial physiological noise. This single-case study employed descriptive analysis to characterize observed patterns.

#### 2.4.1 Pain

Pain was recorded on the visual analog scale, requiring no further analysis.

#### 2.4.2 Task-based connectivity

For functional connectivity, the raw fNIRS data were processed with the NIRS Brain AnalyzIR toolbox (Santosa et al., 2018). The data were down-sampled to 4 Hz, optical density was computed, and the modified Beer–Lambert Law was applied to obtain Oxygenated hemoglobin (HbO) concentrations. Pearson correlation coefficients (r) were calculated using the toolbox’s ‘connectivity’ module, which uses an autoregressive robust correlation function to reduce confounding effects (Huppert, 2016; Santosa et al., 2017). Each correlation coefficient (r-value) represents the relationship between the hemodynamic signals of two channels and serves as a surrogate for functional connectivity. To normalize variance, connectivity values were transformed into Z-scores using Fisher’s transformation (Fisher, 1915).

#### 2.4.3 Hemispheric dominance

Hemispheric dominance was computed using the laterality index (LI) formula (Equation 1). This formula incorporated all the oxygenated hemoglobin values from regions of interest, including the primary motor (M1) and primary sensory (S1) cortices independently, while the premotor cortex (PMC) and supplementary motor area (SMA) were combined for each hemisphere (Borrell et al., 2023a; Seghier, 2008). HbO_L_ and HbO_R_ represent the average oxygenated hemoglobin response over the three one-minute activity tasks, in the left and right hemispheres, respectively. The LI ranges from −1 to +1, where negative values indicate right hemispheric dominance, positive values indicate left hemispheric dominance, and LI values between −0.2 and +0.2 reflect bilateral dominance.


$$\text{Laterality Index}\;(\mathrm{LI})=\frac{\mathrm{ABS}({\mathrm{HbO}}_{L})-\mathrm{ABS}({\mathrm{HbO}}_{R})}{\mathrm{ABS}({\mathrm{HbO}}_{L})+\mathrm{ABS}({\mathrm{HbO}}_{R})}\;$$ (1)

## 3 Results

### 3.1 Pain level

At the pre-TMR interview, the participant reported a pain level of 4 out of 10. Postoperatively, the reported pain level was zero, indicating complete relief.

### 3.2 Movement task functional connectivity

The Fisher transformed matrices (Figures 1C,D) illustrate fNIRS channels correlation strength, reflecting interhemispheric (red dashed box) and intrahemispheric connectivity for each hemisphere before (Figure 1C) and shortly after TMR (Figure 1D). Connectivity was analyzed across the primary motor cortex (M1), premotor/supplementary motor area (PMC/SMA), and primary sensory cortex (S1).

**Figure 2:**
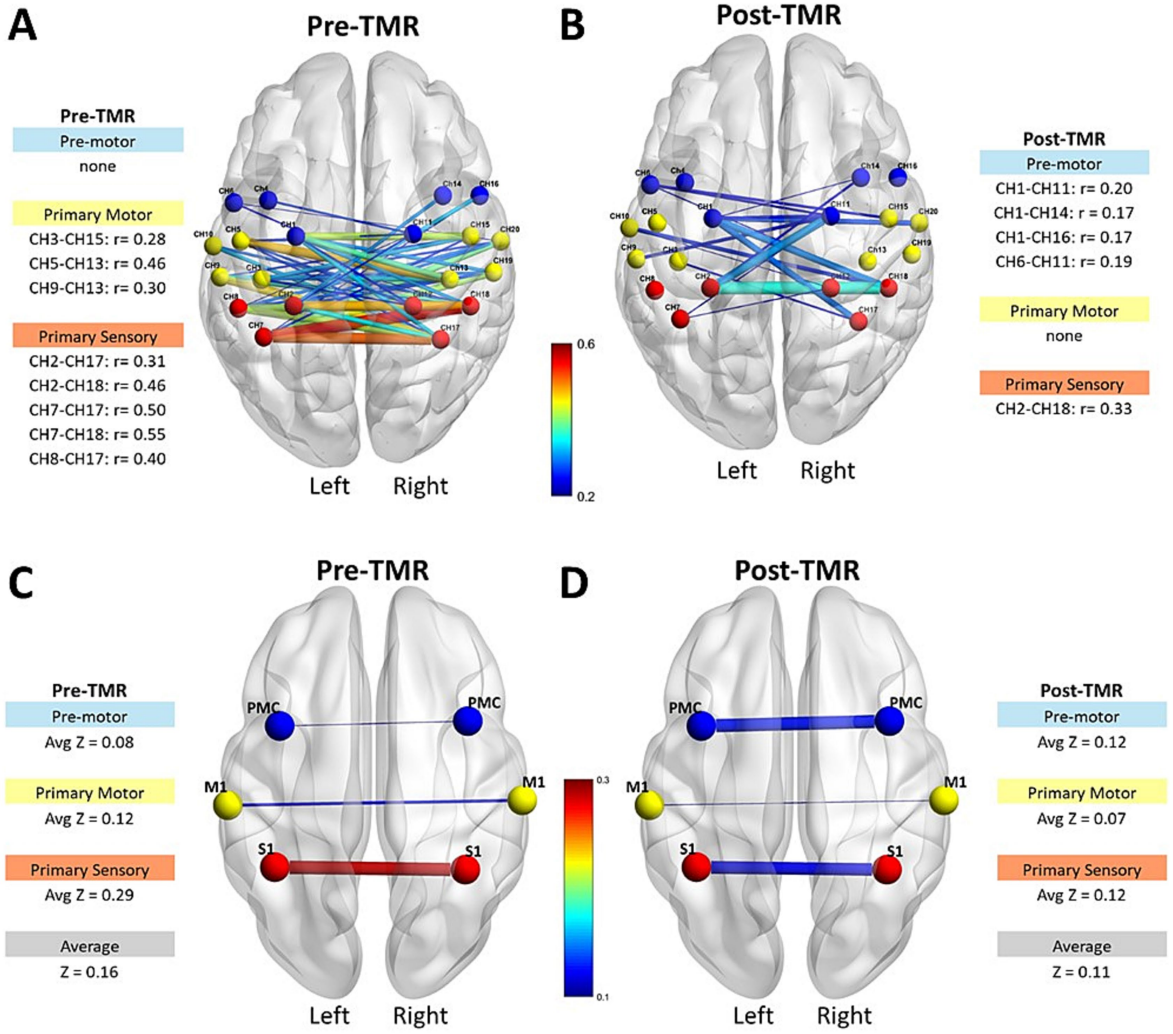
Interhemispheric connectivity strength and Pearson correlation coefficients in motor-related areas pre- and post-TMR intervention. The first row shows connectivity strength and correlation coefficients for M1, PMC/SMA, and S1 regions before **(A)** and after **(B)** the TMR surgery. The second row displays average z values per region and overall hemisphere connectivity before **(C)** and after **(D)** the TMR surgery.

Interhemispheric functional connectivity changed following TMR (Figure 2). The first row illustrates the interhemispheric connectivity strength and the absolute corresponding Pearson correlation coefficients for motor-related channels, M1, PMC/SMA, and S1. Before the intervention (Figure 2A), most interhemispheric channel pairs showed weak correlations (r < 0.3), with moderate correlations (r = 0.4–0.55) in a few S1 channels. Following TMR (Figure 2B), the number of channels between the S1 regions decreased (Baseline = 5, Follow-up = 1), while those between the PMC/SMA increased (Baseline = 0, Follow-up = 4). The second row shows the same interhemispheric channels averaged per region, and overall hemispheric average. Pre-TMR (Figure 2C), the largest average value was between S1areas (z = 0.29), while smaller in M1 (z = 0.12) and PMC/SMA (z = 0.08). After TMR (Figure 2D), there was a decrease in both S1 (z = 0.12) and M1 (z = 0.07), with a slight increase between PMC/SMA (z = 0.12).

**Figure 3:**
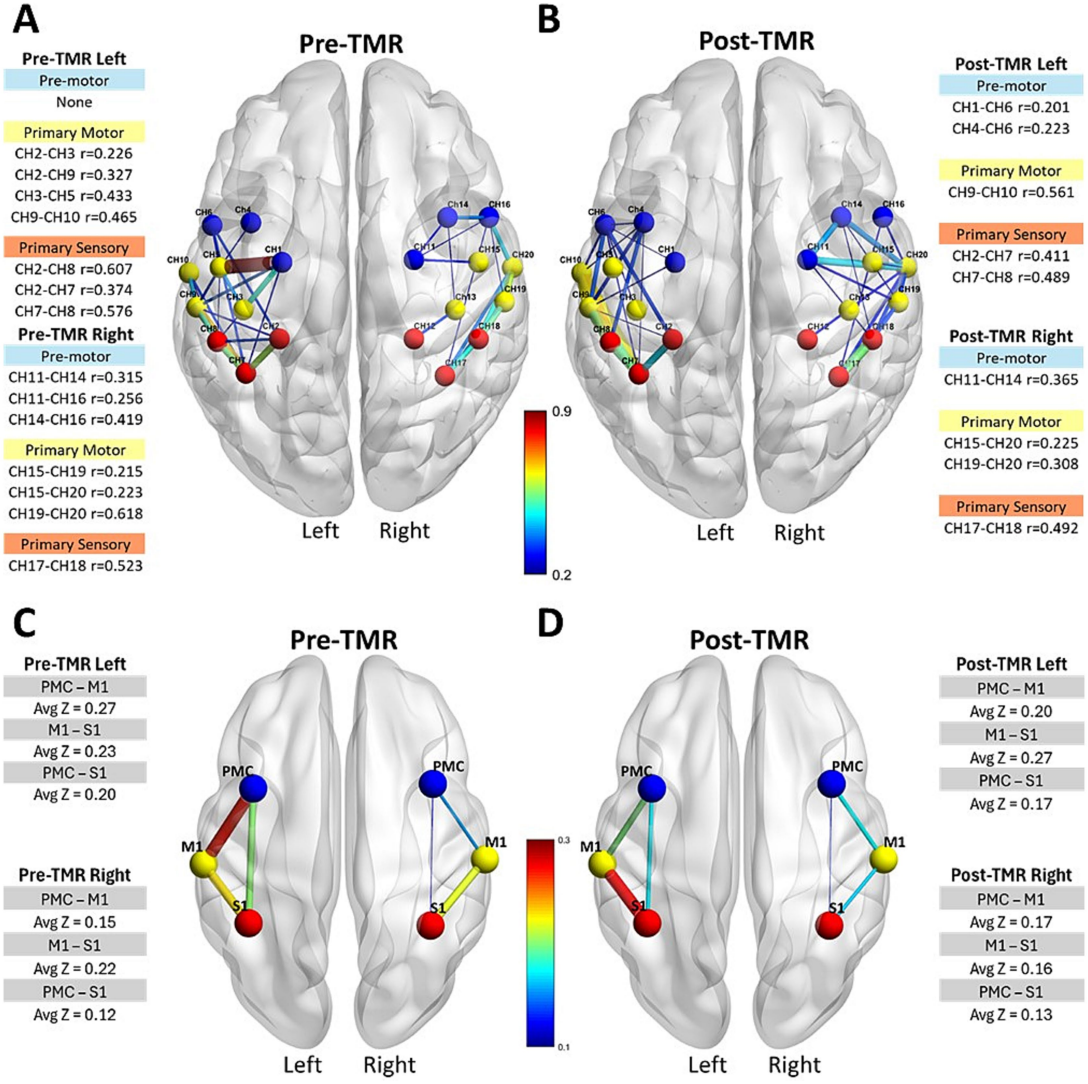
Intrahemispheric connectivity strength and Pearson correlation coefficients in motor-related cortical areas. The first row shows connectivity strength and correlation coefficients for M1, PMC/SMA, and S1 regions within each hemisphere pre-TMR **(A)** and post-TMR Intervention **(B)**. The second row displays average z values per region before **(C)** and after **(D)** the TMR surgery.

Intrahemispheric functional connectivity also changed following TMR (Figure 3). The first row illustrates the intrahemispheric connectivity strength and corresponding absolute Pearson correlation coefficients within motor-related cortical channels, M1, PMC/SMA, and S1 within each hemisphere. Before TMR (Figure 3A), moderately strong correlations (r = 0.4–0.55) were observed in several channels within M1 and S1 in both hemispheres, with the sensory area showing the highest correlation (r = 0.6) on the ipsilateral (left) side. After TMR (Figure 3B), correlations decreased across all sensory channels, with the highest correlation dropping to r = 0.49 in both hemispheres. In the left PMC/SMA, the number of correlated channels increased from zero to two after TMR, though correlations remained weak. (r < 0.3). In the right PMC/SMA, three premotor channels were initially correlated; after TMR, two weakened, while one channel (CH11–CH14) strengthened from r = 0.256 to 0.365. The second row shows average z values for intrahemispheric channels between regions. Prior to TMR (Figure 3C), the left hemisphere (ipsilateral side) showed the highest z value between the PMC/SMA-M1 (z = 0.27), while the z value between the primary sensory and premotor areas was 0.23. On the right hemisphere, the pattern was reversed: the highest z value was between the S1-M1 (z = 0.22), and between PMC/SMA-M1 was 0.15, giving an overall average of z = 0.12, considerably lower than the left hemisphere (Z = 0.20). After TMR (Figure 3D), this pattern shifted. On the left hemisphere, the z value between PMC/SMA-M1 decreased to 0.20, while the z value between M1-S1 increased to 0.27. On the right hemisphere, the values remained lower and showed the reversed pattern.

### 3.3 Hemispheric dominance

**Figure 4:**
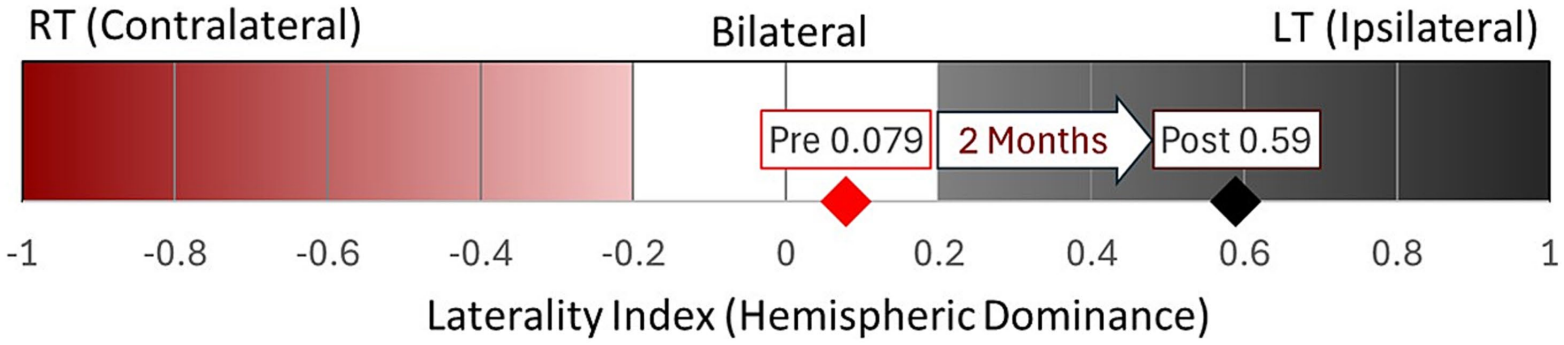
Overall laterality Index within motor-related regions pre- and post-TMR. Post-TMR, activation increased in the primary sensory areas bilaterally, with decreased activation in the contralateral primary motor and premotor regions.

The overall Laterality Index (LI) (Figure 4) before TMR was 0.079, indicating bilateral hemispheric dominance slightly biased ipsilaterally. After TMR, LI increased to 0.59, indicating ipsilateral dominance. Descriptive, region-specific analysis, not illustrated here, suggested that the shift was primarily driven by reduced activation in the contralateral M1 cortex, while other regions remained relatively stable.

## 4 Discussion

### 4.1 Pain

The etiology of PLP remains enigmatic. Traditionally, centrally, it was attributed to somatosensory cortex maladaptive reorganization, with early studies suggesting that increased use of the affected limb could reverse maladaptive plasticity and reduce pain (Flor et al., 1995; Lotze et al., 1999; Nikolajsen and Jensen, 2001). However, recent investigations critically challenge this view (Makin et al., 2013b; Makin and Bensmaia, 2017; Tucciarelli et al., 2024), concluding that cortical changes do not fulfil the criteria for “true reorganization,” which demands the emergence of novel input, novel computation, and distinct connectional fingerprint producing a new functional role. Cortical limb representation remains remarkably stable after sensory loss, and apparent remapping is better explained by the potentiation of pre-existing neural architecture. Given this and the short-term nature of our investigation, interpretations are limited to functional changes, as major structural reorganization, if present, is unlikely within this timeframe. In our participant, the pain was resolved, likely due to neuroma excisions.

### 4.2 Task based connectivity

#### 4.2.1 Interhemispheric functional connectivity

Consistent with previous research in traumatic upper limb loss, interhemispheric connectivity was weak (Hahamy et al., 2015; Makin et al., 2013b). We hypothesized that connectivity would increase after TMR, following pain reduction and subsequent increased affected limb use without a prosthesis, since in people with limb loss, frequent engagement in bimanual tasks strengthens interhemispheric connectivity and pain modulates connectivity patterns (Hahamy et al., 2015; Makin et al., 2013a). Contrary to this, overall interhemispheric connectivity decreased. Reductions were most pronounced in M1 and S1, while areas involved in motor planning (PMC/SMA) (Roland et al., 1980) showed a slight increase.

The corpus callosum inhibits the contralateral hemisphere during unilateral tasks, silencing unused areas. Amputation disrupts this balance, reducing interhemispheric connectivity, especially among individuals reporting higher pain (Sparling et al., 2024). Microstructural alterations, including reduced white matter integrity as indicated by lower fractional anisotropy values in fibers connecting PMC/SMA, are associated with decreased interhemispheric communication in people with limb loss (Bramati et al., 2019; Li et al., 2017) while others show no significant differences compared to controls (Tucciarelli et al., 2024). After 2 months, the small, localized increase in PMC/SMA connectivity may reflect early functional adaptation and possible corpus callosum involvement, although this cannot be determined without structural data. Still, these regions are critical for motor planning and complex motor tasks, and controlling a missing hand without sensory feedback may heavily load these networks, potentially driving the observed connectivity increase (Li et al., 2024; Roland et al., 1980). This is particularly relevant as the participant reported increased limb use and no pain following surgery.

During motor imagery tasks, interhemispheric connectivity in the S1 cortex decreased to zero, consistent with a hand transplantation case study (Piza-Katzer et al., 2007). This may reflect pain relief following neuroma excision and, marginally, transient postoperative medication. In M1, connectivity also decreased, likely reflecting absent functional motor output as newly coapted motor nerves had not yet reinnervated target muscles. Reduced connectivity between M1 and S1 may indicate functional decoupling of the missing limb’s representation from corresponding contralateral areas (Borrell et al., 2023b; Makin et al., 2015). Together with localized PMC/SMA increases, this suggests early-stage functional adaptation after TMR.

#### 4.2.2 Intrahemispheric functional connectivity

No consistent global pattern emerged across intrahemispheric connectivity analyses, with major changes occurring in the ipsilateral (left) rather than the contralateral (right) hemisphere. Before TMR, contralateral hemisphere connectivity was lower than ipsilateral, and both hemispheres showed further reductions after TMR. Across hemispheres, PMC/SMA–M1 connectivity was inversely related to M1–S1 connectivity. Regionally, ipsilateral PMC/SMA–M1 connectivity decreased post-TMR, while M1–S1 connectivity increased; contralateral connectivity remained low, with slight increases in PMC/SMA–M1 and decreases in M1–S1.

In people with lower-limb amputation and phantom sensation but no pain, increased intra-hemispheric connectivity occurs in the deafferented hemisphere during residual and intact limb stimulation, including primary and secondary sensory cortices (S1–S2) and primary motor and premotor areas (Bramati et al., 2019). In our upper-limb TMR patient, only slight increases in contralateral M1–PMC/SMA connectivity were observed post-TMR, with no clear S1–S2 changes. This likely reflects methodological and physiological factors, as no sensory stimulation was used, somatosensory regions were analyzed globally, and measurements occurred early post-TMR, before stabilization. These findings suggest early TMR plasticity emerges in motor circuits, with somatosensory changes appearing later or requiring task-based activation. If replicated, these findings may serve as a neuromarker, guiding early targeted upper motor rehabilitation post-TMR.

### 4.3 Interhemispheric dominance

Before TMR, hemispheric dominance was bilateral, consistent with reports that amputation disrupts interhemispheric connectivity and causes functional decoupling (Borrell et al., 2023b; Makin et al., 2015, 2013b). After TMR, dominance shifted ipsilateral, driven by reduced contralateral M1 activation rather than increased ipsilateral activity. This contrasts with contralateral dominance, typically reported in long-term studies involving repetitive motor practice, reinforcing sensorimotor networks (Serino et al., 2017; Yao et al., 2015). The shift likely reflects passive contralateral suppression due to recently severed efferent pathways, leaving contralateral M1 temporarily disconnected. Comparable reductions in excitability occur in stroke patients (Traversa et al., 1997), and in healthy individuals after short-term immobilization (Ngomo et al., 2012). We interpret this ipsilateral dominance as transient; over time, axonal regrowth and reinnervation should shift towards contralateral dominance. However, axonal regrowth occurs at approximately 1 mm/day (Gordon, 2020) with reliable EMG activity detected at 6 months (Al-Ajam et al., 2022).

## 5 Conclusion

Our short-term post-TMR investigation provides insights into neural plasticity, highlighting changes in both interhemispheric and intrahemispheric connectivity within the sensorimotor cortex and premotor regions. An increase in interhemispheric premotor connectivity appears to be one of the earliest observable signs of cortical adaptation during task-based connectivity assessments. Notably, hemispheric dominance shifts toward the ipsilateral side early in the process, likely due to reduced activation in the primary motor area of the contralateral hemisphere, before newly connected muscles begin to produce EMG signals. This case study demonstrates the potential of functional neuroimaging to assess cortical connectivity changes following TMR, offering a valuable tool for understanding and optimizing motor rehabilitation strategies.

## 6 Limitations

This single-case study has several limitations. Without controls, it is unclear whether changes reflect normalization or TMR-specific adaptations. The participant’s ambidexterity and profession as a painter limit the generalizability of observations to typical TMR patients. Effects of pain reduction, loop closure, and use-dependent plasticity could not be isolated. Additional confounders may have influenced the results, including psychological and mental health factors that affect cortical plasticity.

Methodologically, fNIRS spatial resolution limits analysis to cortical surface activity, and the single motor task may not represent broader changes. Without a longer follow-up, neural activity cannot be linked to functional improvements.

Finally, field gaps complicate interpretation. Limited literature document clear patterns of cortical change following upper limb amputation with clear timelines, inconsistent methodologies, and outcome measures. This hinders the prediction of specific neural adaptation patterns.

Together, these limitations highlight the need for controlled longitudinal studies with larger samples, standardized protocols, and multiple outcome measures.

## Data Availability

The raw data supporting the conclusions of this article will be made available by the authors, without undue reservation.
